# Prenatal Depression, Breastfeeding, and Infant Gut Microbiota

**DOI:** 10.3389/fmicb.2021.664257

**Published:** 2021-07-30

**Authors:** Nicole Rodriguez, Hein M. Tun, Catherine J. Field, Piushkumar J. Mandhane, James A. Scott, Anita L. Kozyrskyj

**Affiliations:** ^1^Department of Pediatrics, University of Alberta, Edmonton, AB, Canada; ^2^HKU-Pasteur Research Pole, Li Ka Shing Faculty of Medicine, School of Public Health, The University of Hong Kong, Hong Kong, China; ^3^Department of Agricultural, Food and Nutritional Science, University of Alberta, Edmonton, AB, Canada; ^4^Dalla Lana School of Public Health, University of Toronto, Toronto, ON, Canada

**Keywords:** prenatal depression, breastfeeding, birth mode, infant, gut microbiota, gut immunity

## Abstract

Depressive symptoms are common during pregnancy and are estimated to affect 7–20% of pregnant women, with higher prevalence found in those with a prior history of depression, in ethnic minorities, and those with increased exposure to stressful life events. Maternal depression often remains undiagnosed, and its symptoms can increase adverse health risks to the infant, including impaired cognitive development, behavioral problems, and higher susceptibility to physical illnesses. Accumulating research evidence supports the association between maternal physical health elements to infant gut health, including factors such as mode of delivery, medication, feeding status, and antibiotic use. However, specific maternal prenatal psychosocial factors and their effect on infant gut microbiota and immunity remains an area that is not well understood. This article reviews the literature and supplements it with new findings to show that prenatal depression alters: (i) gut microbial composition in partially and fully formula-fed infants at 3–4 months of age, and (ii) gut immunity (i.e., secretory Immunoglobulin A) in all infants independent of breastfeeding status. Understanding the implications of maternal depression on the infant gut microbiome is important to enhance both maternal and child health and to better inform disease outcomes and management.

## Introduction

The World Health Organization (WHO) lists depression as the leading cause of disease burden for women of reproductive age (Davalos et al., 2012). Depression before and after birth is often accompanied by symptoms of sadness and anxiety, anhedonia, appetite loss, sleep disturbance, confusion, and mood lability (Bernard-Bonnin et al., 2004). An estimated 18.4% of women experience prenatal depression, with 12.7% having major depressive episodes, and 13% experiencing postpartum depression. Women in their reproductive years are especially at high risk for major depression, with increased risk present during pregnancy or within the first 12 months post-delivery (Figueiredo et al., 2014). When left undetected, maternal depression results in reduced mother-child quality interactions, including reduced breastfeeding, impaired response to the infant’s hunger cues, and less contingent stimulation. Prenatal depression is a significant contributor to shorter breastfeeding duration (Dias and Figueiredo, 2015).

In addition to its negative impact on maternal health, prenatal depression can have long term consequences on children’s physical and mental health, and cognitive and socio-emotional development (Bernard-Bonnin et al., 2004). Depression alters the intrauterine environment, including elevation in circulating cortisol, which can negatively affect the developing fetus (Lewis et al., 2015). Various epidemiological studies suggest the association of maternal depression and the development of a compromised infant immune system susceptible to illnesses including asthma, allergy, and other atopic diseases (Smejda et al., 2019). Fetal exposure to prenatal depression predicts elevated inflammatory biomarkers at age 25. Prenatal depression can also extend its influence to the critical “window of opportunity” during the infant’s first year of life and the beginning stages of gut microbiota development when it is most sensitive to perturbation (van den Elsen et al., 2019).

Several factors have been identified as key to shaping early microbiota composition and function, including birth mode, antibiotic use, and infant nutrition (Milani et al., 2017). Next to birth mode, breastfeeding has the most critical influence in young infants. Breast milk shapes the gut microbiota composition of infants by providing nutrients for bacterial growth (Bravi et al., 2016; Moossavi et al., 2019). Infants who are partially breastfed and even those who receive small amounts of formula supplementation display significant shifts in their gut microbial composition (Forbes et al., 2018). Additionally, breastfeeding also shapes infant immune development to support oral tolerance induction and allergy prevention (van den Elsen et al., 2019).

Many studies support the importance of early-life factors, including maternal and infant factors, in establishing nascent gut microbiota that consequently contribute to infant nutrient acquisition, pathogen exclusion, immune system regulation, and other health and developmental outcomes. This brief review aims to present evidence from the literature that examines maternal prenatal depression’s relationship with infant gut microbiota and immunity, taking into account maternal prenatal diet and infant diet.

## Prenatal Depression Affects Infant Gut Microbial Composition Dependent on Breastfeeding

To understand the link between maternal depression, breastfeeding, and infant gut microbiota, it is essential to consider the decision-making process that women undergo when choosing to breastfeed. Breastfeeding behavior has two stages (Meedya et al., 2010). First is the *intention* or the decision to breastfeed, which is shaped by sociodemographic, clinical, and psychosocial factors; and second is *initiation*, which is dependent on the intention, as well as lactation coaching, birth mode, and perinatal complications. The overwhelming majority of women make decisions on whether to breast or formula feed during the prenatal period; intention is one of the strongest predictors of breastfeeding initiation (Bogen et al., 2010). A comprehensive systematic review revealed that prenatal depression may or may not reduce breastfeeding intention (Dias and Figueiredo, 2015). It does not appear to reduce breastfeeding initiation, pointing to the success of lactation coaching. However, depression during pregnancy predicts shorter breastfeeding duration. Hence, prenatal depression may make no difference on whether a woman intends to and/or initiates breastfeeding, but it is certainly a factor in whether she continues to breastfeed. Lastly, prenatal depression appears to have a more substantial impact on breastfeeding duration than postnatal depression (Dias and Figueiredo, 2015).

**FIGURE 1:**
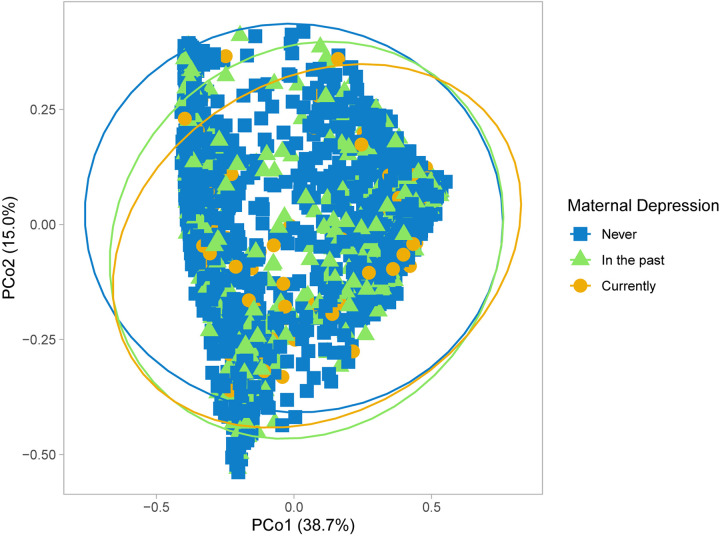
Microbial community structure of infant gut microbiota according to maternal prenatal depression. Principal coordinate analysis performed using Bray-Curtis dissimilarity matrices.

Building on previous studies of maternal perinatal depression and the infant gut microbiome (Zijlmans et al., 2015), we compared whole gut microbiota community composition in infants at mean age 3.7 months according to maternal depression status in the CHILD Cohort Study (Box 1). Differences were found in Bray-Curtis measure of microbial beta-diversity between infants of mothers with and without prenatal depression (Figure 1 and Table 1; Beals, 1984). Notably, these observed gut microbial community differences were independent of breastfeeding status, and of many maternal and household factors known to influence infant gut microbiota, including breastfeeding difficulty. There were interactions in maternal mood differences according to breastfeeding status that will be discussed later. Further, infant gut microbial abundance differed according to maternal history of depression, classified as during pregnancy, in the past and never (Figure 1 and Table 2). Gut bacteria in the families of the phylum, Firmicutes families – Erysipelotrichaceae, Lachnospiraceae, and Ruminococcaceae were more abundant in infants of mothers experiencing depression during pregnancy versus infants whose mothers never had depression and those with depression in the past. Based on pairwise comparisons of abundance, only Ruminococcaceae and Lachnospiraceae showed a statistically significant difference between all three depression categories (*p* < 0.01), including enrichment in infants whose mothers had depression in the past versus those who did not. These butyrate-producing bacteria are strict anaerobes that normally become more abundant in later infancy. Many have been found to be elevated in the gut of adults with depression and of mice following fecal transplantation from adults with depression, although not uniformly so (Barandouzi et al., 2020).

**Box 1. Methods.**

**Study population**

The present study involved a subsample of 996 term infants from three study sites (Edmonton, Vancouver, and Winnipeg) of the CHILD birth cohort (www.childstudy.ca). Mothers were mainly of Caucasian ethnicity (75.3%), between the ages of 30 and 34 (39.9%), and were generally healthy. One quarter of women reported depression during pregnancy or in the past. Most infants were born vaginally (74.7%), and 11.3% by elective and 14% by emergency cesarean section. At 3–4 months, 43.2% of infants were exclusively breastfed, including 29.4% exclusively breastfed since birth and 13.8% who briefly received formula in hospital. An additional 34.3% were partially breastfed and 22.4% were not breastfed at all. Cesarean delivery rates increased from 20 to 29% as exclusivity of breastfeeding declined, but were highest in exclusively breastfed infants receiving some formula after birth (31.5%).

**Study measures**

For the purposes of this study, the term breastfeeding is used to describe feeding the infant breast milk, whether at the breast or from the bottle. At 3 months postpartum, mothers completed questionnaires reporting on breastfeeding status and the introduction of formula. Breastfeeding status was classified into four groups as exclusive (breast milk only), exclusively breastfed after hospital, partial (breast milk and formula), and none (formula only). Patient chart reviews were used to classify infants into exclusively breastfed “after hospital” if they briefly received formula in hospital but were thereafter exclusively breastfed following hospital discharge. Breastfeeding status was determined for the same date as fecal sample collection. Candidate confounding factors were selected from a literature review. They included covariates such as mode of delivery, parity, gestational diabetes, infant sex, birth weight, and hospital-administered antibiotics to the mother or neonate that were obtained from hospital records. Mothers also completed standardized questionnaires during pregnancy and 3 months postpartum for information related to maternal characteristics such as race (Asian, Caucasian, First Nations, and Other), age, post-secondary education (Yes/No), smoking and pre-existing conditions (i.e., recurrent urinary tract infections) or other factors such as presence of siblings, pets in household or cleaning product usage. Additionally, the Healthy Eating Index was used to assess the quality of maternal diet (Guenther et al., 2013). Fecal stool samples were collected at a home visit at 3–4 months (mean ± SD: 3.7 ± 1.0) and were transported to the laboratory on ice and stored at −80°C until processing. DNA was extracted using the QIAamp DNA Stool Mini Kit (Qiagen Inc., Valencia, CA, United States). The 16S ribosomal RNA (rRNA) V4 region was amplified by primers 515f (TATGGTAATTGTGTGCCAGCMGCCGCGGTAA) and 806r (AGTCAGTCAGCCGGACTACHVGGGTWTCTAAT). PCR amplicons were pooled and multiplexed (48 or 96 samples per run) and sequenced on an Illumina MiSeq (San Diego, CA, United States) to generate 2 × 150 bp. The QIIME (v 1.8.0) platform was used to analyze 16S rRNA amplicon data (Desantis et al., 2006; Caporaso et al., 2010). Briefly, reads were assembled, demultiplexed and clustered at 97% using USEARCH (v 6.1). Taxonomic classification of sequence clusters was performed using the Greengenes reference database (v 13.8); non-bacterial sequences clustered at 97% similarity, and singletons were removed. The final dataset included a total of 265,095,597 sequences (median 235,623 per sample, range 13,134–833,392), corresponding to 939 unique OTUs. Data were rarefied to 13,000 sequences per sample and summarized at various taxonomic ranks.

**Statistical analysis**

Microbiota community structures were visualized using principal coordinate analysis (PCoA). Total microbial beta-diversity, measured by the Bray-Curtis method, was tested by permutational analysis of variance (PERMANOVA) with 999 permutations using the adonis function from the R “vegan” package (Anderson, 2001). To reduce confounding bias, covariates were added to models in a sequential order of most to least variance explained (i.e., F-model). Analyses were performed in R (version 3.3.3; R Development Core Team). Taxon median relative abundance was compared by non-parametric Kruskal–Wallis tests and *post hoc* Dunn’s tests with false discovery rate (FDR) correction for multiple comparisons.

**TABLE 1:** Univariable and multivariable PERMANOVA analysis of infant gut microbiota at 3–4 months in the CHILD cohort.

	**Univariable**		**Multivariable^a^**	
	**Pr(>F)^b^**	**R2^c^**	**Pr(>F)^b^**	**R2^c^**
**All infants^d^**				
Prenatal depression	**0.001**	0.0057	**0.006**	0.0048
Birth mode	**0.001**	0.0523	**0.001**	0.0597
Breastfeeding status	**0.001**	0.0457	**0.001**	0.0448
**Exclusive breastfeeding from birth up to 3–4 months**				
Prenatal depression	0.706	0.0020	0.566	0.0031
Birth mode	**0.001**	0.0701	**0.001**	0.0759
**Exclusive breastfeeding at 3–4 months but not in hospital after birth**				
Prenatal depression	0.701	0.0043	0.633	0.0058
Birth mode	**0.001**	0.0756	**0.016**	0.0577
**Partial breastfeeding at 3–4 months**				
Prenatal depression	**0.003**	0.0133	**0.023**	0.0111
Birth mode	**0.001**	0.0602	**0.001**	0.05801
**No breastfeeding at 3–4 months**				
Prenatal depression	**0.040**	0.0116	**0.022**	0.0274
Birth mode	**0.001**	0.0510	**0.001**	0.0971

*^*a*^Adjusted for maternal age, race, pre-pregnancy BMI, prenatal diet (healthy eating index/HEI), recurrent UTI, and smoking, birth mode, infant sex, antibiotics, length of hospital stay, BF difficulty medication, older siblings, dog in household, and study site.*

*^*b*^*P*-value corresponds to the beta-coefficient and is for each of the variables.*

*^*c*^*R*^2^ value is specific to each of the covariates.*

*^*d*^Association of prenatal depression in all infants (i.e., not stratified by breastfeeding status at 3–4 months).*

*^*e*^Bolded values are statistically significant.*

**TABLE 2:** Median abundance of bacterial taxa in infant gut microbiota at 3–4 months according to reported status of maternal depression.

**Taxon**	**Maternal prenatal depression**				
	**Never**	**In the past**	**Currently**	**pFDR**	**Pairwise pFDR**
Actinobacteria	12.74	11.94	9.48		
Actinomycetaceae	0.21	0.09	0.33		
Bifidobacteriaceae	11.99	11.23	8.74		
Coriobacteriaceae	0.42	0.50	0.37		
Micrococcaceae	0.12	0.11	0.04		
Bacteroidetes	29.46	32.46	33.15		
Bacteroidaceae	26.62	30.34	28.36		
Porphyromonadaceae	2.17	1.56	2.16		
Firmicutes	28.28	30.83	33.72		
Clostridiaceae	4.21	4.05	2.02		
Enterococcaceae	0.16	0.16	0.10		
Erysipelotrichaceae	0.95	0.59	0.96		bc
Lachnospiraceae	8.01	8.71	9.81		abc
Ruminococcaceae	1.87	1.89	4.03	*	abc
Streptococcaceae	2.05	2.20	0.90		bc
Veillonellaceae	10.30	12.42	15.06		
Proteobacteria	27.38	22.40	20.28	*	ab
Enterobacteriaceae	25.67	20.14	18.42	*	ab
Pasteurellaceae	0.85	0.84	0.61		

Taxa with median abundance >0% in 3–4 months microbiota. Overall comparisons by rank-based non-parametric Kruskal–Wallis test with FDR correction for multiple comparisons; *p < 0.01. Pairwise comparisons by Dunn’s *post hoc* tests for multiple comparisons: ^*a*^Never/In the past; ^*b*^Never/Currently; ^*c*^In the past/Currently.

Interestingly, CHILD study infants born to women with prenatal or history of depression had significantly fewer Proteobacteria in their gut microbiota, specifically the Enterobacteriaceae, than infants whose mothers never had depression (Table 2). Typically, Proteobacteria peak in abundance during early infancy — a phenomenon called the “Proteobacteria bloom,” and then slowly decline after the first 3 years of life as gut microbiota start to resemble that of an adult (Shin et al., 2015). This Proteobacterial bloom plays a crucial role in infant immune mechanisms, including homeostasis and tolerance to environmental pathogens, and prepares the infant gut for colonization by strict anaerobes in later infancy. As summarized by Campos-Rodríguez et al. (2013), evidence is accumulating from several studies that maternal prenatal depression affects phylogenetic diversity of second trimester gut microbiota, and in turn, gut microbial colonization of offspring. More recently, Hu et al. (2019) found psychosocial stress during pregnancy, specifically anxiety, also to be associated with a less diverse microbial community in meconium (first stool) of the newborn (Hu et al., 2019). Since passage of meconium predates breastfeeding in many infants, their results point to a causal pathway for prenatal depression. It is striking then, that CHILD study results were consistent with theirs, on the correlation between higher pregnancy-related anxiety with lower levels of meconium Enterobacteriaceae. Further, a small study of longitudinally collected fecal samples after vaginal birth found enrichment with some enterobacterial genera but a reduction in other enterobacteria over the first 4 months of life when maternal stress, anxiety or cortisol levels were high in the last trimester of pregnancy; lactobacilli and bifidobacteria were also less abundant (Zijlmans et al., 2015). Thus, evidence is accumulating that prenatal depression disrupts healthy development of offspring gut microbiota months after birth. This process may be initiated by the transfer of a suboptimal maternal microbiome to the newborn, which may promote the same microbiota that are elevated in women with depression (Barandouzi et al., 2020).

Ample research supports the importance of breast milk to the establishment of gut microbiota (Williams et al., 2019) and provision of essential human milk oligosaccharides for microbial growth (Moossavi et al., 2019). Mother’s milk also carries with it microbiota and metabolites derived from multiple sources, including the breast’s surface, lactiferous ducts, or from the maternal gut (Dieterich et al., 2013). Within the first 6 months of life, breastfeeding is only second to birth mode as a substantial and independent determinant of gut microbial composition (Madan et al., 2016; Yang et al., 2019). Next to birth mode, breastfeeding status explained the next highest variation in community diversity (r-squared, 4.5%) in CHILD study infants at mean age 3.7 months (Table 1). Yet, independent of 15 adjusting covariates, including infant feeding status, prenatal depression ranked 4th to explain 0.5% of the variation in gut microbial diversity among all infants.

It is also important to know how prenatal depression impacts the gut microbiota of infants within feeding groups (e.g., exclusive, partial, and no breastfeeding). Similar gut microbial dysbiosis has been reported in 3–4 months old infants following maternal prenatal stress in the presence or absence of breastfeeding (Zijlmans et al., 2015). In the CHILD study, no gut microbiota compositional differences were found by maternal prenatal mood status in fully-breastfed infants at 3–4 months, in whom the major determinant of gut microbial diversity was birth mode (Table 1). In contrast, prenatal depression was associated with statistically significant changes to total microbial diversity of infants who were not exclusively breastfed (Table 1). Enrichment with Lachnospiraceae and Ruminococcaceae, and depletion of Enterobacteriaceae are characteristic of 3–4 months gut microbiota during partial or full formula-feeding when compared to exclusive breastfeeding, and independent of birth mode (Forbes et al., 2018). Prenatal depression further enhanced the abundance of Lachnospiraceae and Ruminococcaceae in CHILD study infants (Table 2). Importantly, next to cesarean birth (r^2^ up to 9.7%), prenatal depression ranked 2nd in explaining the variation in gut microbial diversity in the partially (1.1%) and fully formula-fed (2.7%) groups (Table 1).

Shifts in the normal development of infant gut microbiota, such as the premature depletion of Proteobacteria or enrichment of butyrate-producers, increase risk for gastrointestinal and allergic diseases, and future overweight (Milani et al., 2017; Forbes et al., 2018; Korpela and de Vos, 2018; Tun et al., 2018). There is a growing appreciation of signaling pathways of the “gut-brain axis” that involve gut microbiota. Since the prenatal and postnatal periods are important phases of development for both the brain and gut, significant potential exists for maternal distress to have long-term effects on both gut microbiota and neurodevelopment (Codagnone et al., 2019). In summary, human evidence is amassing on the detrimental impact of prenatal depression on gut microbiota in offspring. Since prenatal depression results in shorter breastfeeding duration, and because breastfeeding is a strong predictor of microbiome composition, the evidence implies that prenatal depression harms the infant gut microbiome by reducing duration of breastfeeding. In this section, we also pointed to the limited research on differential impacts of maternal depression according to breastfeeding status, which presents additional risk of adverse outcomes in infants not exclusively breastfed in early life.

## Prenatal Depression Affects Infant Gut Immunity Independent of Breastfeeding

The early establishment of the infant gut microbiome has a significant influence on postnatal development of the gut mucosal immune system, and early infancy is a critical window of influence for both systems (Milani et al., 2017). The innate and adaptive immune systems both develop in concert with gut microbiota, and both are required to achieve host-gut microbiota homeostasis. Secretory IgA (sIgA) is a mucosal immunoglobulin of the adaptive immune system that acts as the first line of defense against invading pathogens by coating bacteria (Corthésy, 2013). It also binds to members of the gut microbiota, promoting homeostasis by preventing overgrowth by a single species. Since infants are unable to produce sIgA in significant amounts during the early days of postnatal life, they are highly dependent on their mother’s breast milk as a source for sIgA (Maruyama et al., 2009). Suboptimal binding of Proteobacterial microbiota to sIgA has been associated with gastrointestinal conditions such as necrotizing enterocolitis in preterm infants, and even future allergic disease (Dzidic et al., 2017; Hornef and Torow, 2020).

Gut microbiota and sIgA binding are selective, such that several members of the Firmicutes (e.g., lactobacilli, Lachnospiraceae, and Ruminococcaceae) are preferentially bound to gut sIgA (Macpherson et al., 2018). Specific gut microbiota, like the lactobacilli, and their metabolites can also promote the infant’s own production of sIgA by gut mucosal cells and increase sIgA binding to microbiota (Kukkonen et al., 2010; Kim et al., 2016). Inverse associations between *Clostridium difficile* colonization and gut sIgA levels in young infants have been reported (Drall et al., 2019). As such, an imbalance in infant gut microbiota from the depletion (e.g., lactobacilli or enterobacteria) or enhancement of specific microbiota (e.g., members of the Lachnospiraceae) subsequent to prenatal depression has capacity to affect sIgA binding and/or production with further disruption to host-gut microbiota homeostasis. Animal experimental models have confirmed a causal association between psychological stress and adaptive gut immunity. When young mice are exposed to repeated restraint stress (stress due to immobilization), significantly lower intestinal sIgA levels and number of IgA-producing cells in intestinal mucosa are observed (Campos-Rodríguez et al., 2013). The natural stress experienced by mice when they change cages or social groups has also been found to lower fecal sIgA levels. Posited pathways for prenatal distress include greater transmission of glucocorticoids to the developing fetus that reduces the number of IgA-producing cells (Campos-Rodríguez et al., 2013; Lewis et al., 2015). Equally plausible are postnatal sIgA changes secondary to gut microbial dysbiosis from prenatal distress. More recently, restraint stress in a murine model raised the extent of gut IgA binding of microbiota like the Lachnospiraceae; this result was replicated in fecal samples from adults with irritable bowel syndrome, a condition often aggravated by stress (Rengarajan et al., 2020).

Kang et al. (2020) published the first human report regarding the independent association between maternal depressive symptomatology during pregnancy and compromised gut immunity in offspring. This recent study which examined infant fecal sIgA concentrations in relation to maternal depression trajectories, revealed that infants born to mothers in the prenatal trajectory (high depressive symptoms scores primarily during pregnancy) were twice more likely to have lower sIgA concentrations than infants of mothers with low symptom scores. At 4–8 months of age, the reduction in fecal sIgA concentrations among infants of mothers in the prenatal trajectory amounted to a large effect size of 0.53. Importantly, in the presence of prenatal depression, they were equally likely to be low in infants not breastfed, who fully depend on self-production of sIgA, and in exclusively breastfed infants. The latter results are consistent with previous findings by Kawano and Emori (2015) in which maternal psychological states affect immune properties of breast milk. This breast milk study demonstrated that women who scored highly on measures of anxiety, depression, and anger tended to have lower sIgA concentrations in their milk.

In summary, maternal depression during pregnancy can compromise offspring adaptive immunity through direct actions of cortisol on fetal development of IgA-producing cells and indirectly, through postnatal changes to infant gut microbiota and altered sIgA production or binding. Consequently, lowered gut sIgA concentrations or abundance of IgA-stimulating gut microbiota can impair microbe-sIgA interactions, increasing the risk of *C. difficile* colonization and allergic disease. Even if prenatal depression does not alter gut microbiota in exclusively breastfed infants (see above section), *in utero* action to reduce the number of IgA-producing cells has real potential to affect IgA-gut microbiota binding in exclusively breastfed infants. Finally, women experiencing distress have lowered sIgA amounts in breast milk and they are less likely to breastfeed for a longer duration.

## Prenatal Depression Influences the Infant’s Gut Microbiome Independent of Prenatal Diet

To maintain a healthy pregnancy, adequate nutrition is needed to nourish both mother and fetus. A narrative review by Boutté et al. (2021) confirmed that worldwide, women with depression or stress during pregnancy eat an unhealthy diet, high on fat, and low in fruits and vegetables. Aspects of the prenatal diet related to fat consumption have been associated with changes to pregnancy and infant gut microbiota (Chu et al., 2016; Mandal et al., 2016). Notably, greater self-reported fruit intake during pregnancy has been linked to enhanced neurodevelopment in the CHILD study (Bolduc et al., 2016).

In the above-mentioned CHILD study, a “healthy” prenatal diet (i.e., an HEI score of above 80) was strongly associated with gut microbial diversity in infants at 3–4 months (*r*^2^, 0.37%, *p* = 0.009). This univariate association remained only within non-breastfed infants, in whom prenatal diet explained a greater percentage of the variation in beta-diversity (*r*^2^, 1.08%, *p* = 0.04). Prenatal diet associations with infant gut microbiota were lost altogether in multivariate models that included prenatal depression (Table 1). These results demonstrate that maternal depression influences the infant gut microbiome even following adjustment for prenatal diet quality. They also indicate that a healthy maternal prenatal diet is especially important to infants who are not breastfed, the benefits of which may be affected by depression during pregnancy.

## Conclusion

Evidence is accumulating on the association between maternal prenatal depression and gut microbial diversity in early infancy. Characteristic of the altered gut microbial community structure and important to the infant’s adaptive immunity are disruptions in the typical Proteobacterial bloom, and enrichment with microbiota in the families *Ruminococcaceae* and *Lachnospiraceae*. The latter is seen in infants who develop overweight. We reported new evidence that prenatal depression-related differences in microbial diversity are more likely to manifest in infants with partial or absence of breastfeeding. On the other hand, prenatal depression appears to affect sIgA independent of infant feeding type. Future study is needed to more fully characterize gut microbiota changes at the genus level, and to include viral and fungal, and other communities as well. This micro-level characterization also requires testing for metabolism end-products to determine the specific pathways by which maternal depression affects the infant gut microbiome/virome/mycobiome and future health.

Breastfeeding has many psychological benefits; our review suggests that breastfeeding can be protective against the impact of maternal depression on infant gut microbiota. It also emphasizes the importance of pregnancy depression screening to identify women at risk for breastfeeding cessation since type of infant feeding strongly influences gut microbial composition. The review also underscores the importance of prenatal counseling on depression and dietary intake to promote future gut health of the newborn. Finally, obtaining evidence on the detrimental impact of prenatal depression on infant gut microbiota is critical to inform strategies that identify and timely target women with depression during pregnancy, as their infants will benefit from breastfeeding coaching to prolong its duration.

## Ethics Statement

The studies involving human participants were reviewed and approved by the University of Alberta Health Research Ethics Boards. Written informed consent to participate in this study was provided by the participants’ legal guardian/next of kin.

## Author Contributions

AK contributed to the conception and design of the study. HT, CF, PM, and JS organized the database. NR wrote the first draft of the manuscript. All authors contributed to manuscript revision, read, and approved the submitted version.

## Conflict of Interest

The authors declare that the research was conducted in the absence of any commercial or financial relationships that could be construed as a potential conflict of interest.

## Publisher’s Note

All claims expressed in this article are solely those of the authors and do not necessarily represent those of their affiliated organizations, or those of the publisher, the editors and the reviewers. Any product that may be evaluated in this article, or claim that may be made by its manufacturer, is not guaranteed or endorsed by the publisher.
